# CircMETTL3 Inhibits H_2_O_2_‐Induced Senescence, Oxidative Stress, and DNA Damage in ARPE‐19 Cells via miR‐100/BMPR2 Axis

**DOI:** 10.1155/joph/3194421

**Published:** 2026-08-20

**Authors:** Xiangyang Xin, Xin Zhao, Feng Ling, Liru Qin, Xinyue Liu, Changhe Liu

**Affiliations:** ^1^ Department of Ophthalmology, Baotou Central Hospital, Baotou 014040, Inner Mongolia, China, btzxyy.com; ^2^ Department of Ophthalmology, The Third Affiliated Hospital of Inner Mongolia Medical University (Inner Mongolia Baogang Hospital), Baotou 014010, Inner Mongolia, China; ^3^ Tianjin Key Laboratory of Retinal Functions and Diseases, Branch of National Clinical Research Center for Ocular Disease, Eye Institute and School of Optometry, Tianjin Medical University Eye Hospital, Tianjin, China, tmuec.com

**Keywords:** ARPE-19 cells, BMPR2, circMETTL3, H_2_O_2_, miR-100

## Abstract

**Background:**

Age‐related macular degeneration (AMD) is a common degenerative eye disease that eventually leads to irreversible vision loss. CircRNAs have received increasing attention for their regulatory role in AMD. In this study, whole transcriptome sequencing identified differentially expressed circRNA (circMETTL3) in AMD. Previous studies have unlocked the potential mechanism of circMETTL3 in cancer, but its role in AMD has not been studied.

**Methods:**

ARPE‐19 cells treated with H_2_O_2_ were used as the AMD cell model. The senescence, oxidative stress, and DNA damage of ARPE‐19 cells were determined by SA‐β‐gal staining, DCFH‐DA staining, and IF assay. The levels of RPE‐specific markers or mRNA levels were assessed using western blot assay. The potential mechanism of circMETTL3 was investigated by luciferase reporting assay and RIP assay.

**Results:**

CircMETTL3 was revealed to decrease in AMD cell models. The addition of circMETTL3 decreased SA‐β‐gal staining and ROS production and facilitated cell viability in H_2_O_2_‐treated ARPE‐19 cells. Moreover, γH2AX and KRT18 levels were suppressed, and TJP1, BEST1, and CTNNB1 protein levels were increased by circMETTL3 addition. In addition, circMETTL3 could bind to and negatively regulate miR‐100. Overexpression of miR‐100 exacerbated senescence, oxidative stress, and DNA damage in H_2_O_2_‐treated ARPE‐19 cells, which reversed the effects of circMETTL3. Furthermore, BMPR2 was targeted by miR‐100. Overexpression of BMPR2 inhibited H_2_O_2_‐induced cell damage in ARPE‐19 cells, which reversed the effects of miR‐100.

**Conclusions:**

In sum, these findings demonstrated that the circMETTL3/miR‐100/BMPR2 axis plays a vital regulatory role in AMD development.

## 1. Introduction

Age‐related macular degeneration (AMD), a progressive retinal degeneration characterized by the loss of central vision, is an important cause of visual impairment in the elderly [1]. With the increase of the aging population, the incidence of AMD is gradually increasing, affecting the quality of life of patients and increasing the social burden [2]. AMD is mainly divided into dry and wet forms; about 87% of AMD patients belong to the dry type of cases. Intravitreal injections of anti‐VEGF drugs have been shown to be effective against wet AMD [3]. However, there is still a lack of effective treatments for dry AMD. Retinal pigment epithelial (RPE) cell injury is an important cause of visual impairment in patients with dry AMD [4]. Therefore, understanding the underlying mechanism of RPE injury is important for developing prevention and treatment strategies for patients with dry AMD.

Aging is a time‐dependent degenerative process associated with a range of diseases, including neurodegeneration [5], cardiovascular disease [6], and osteoarthritis [7]. Aging is also a vital cause of visual impairment. The photoprotection of RPE decreases with age. In addition, RPE undergoes typical structural changes during aging, including drusen accumulation, apical microvilli atrophy, and progressive cell loss [8]. Age‐related changes in cells, such as DNA damage, oxidative stress, and telomere erosion, are powerful triggers for inducing aging [9]. Understanding the mechanism of RPE senescence might be important for the prevention and treatment of AMD.

Over the past few decades, noncoding RNAs (ncRNAs) have made up the majority of the human transcribed genome, regulating cell physiology and shaping cell function [10]. Circular RNA (circRNA) is a type of ncRNA characterized by a covalently closed circular structure [11]. CircRNAs are resistant to RNA exonuclease so that they can be more stable in tissues and cells [12]. Previous studies have indicated that circRNAs played a wide range of functional roles in various biological processes and cellular activities [13]. Lately, abnormal expression of circRNAs has been demonstrated in some retinopathy. For example, circRNA DMNT3B, as a miR‐20‐5p sponge, was downregulated in the epiretinal membrane in patients with diabetic retinopathy, and this downregulation is closely associated with retinal vascular dysfunction [14]. Among the numerous circRNAs, circMETTL3 has attracted considerable attention due to its regulatory functions in various pathological contexts. CircMETTL3 is derived from the METTL3 gene, which encodes a key component of the N6‐methyladenosine (m⁶A) methyltransferase complex. Accumulating evidence indicates that circMETTL3 is involved in tumor progression, functioning as a microRNA sponge to regulate downstream oncogenic or tumor‐suppressive pathways. For example, circMETTL3 has been reported to promote breast cancer proliferation and metastasis by sponging miR‐31‐5p [15]. In addition, circMETTL3 is involved in the regulation of inflammation in nonocular systems. For instance, circMETTL3‐156aa remodels glycolytic metabolism in macrophages, promotes M1 polarization, and induces cytokine storms [16]. However, the expression pattern and functional significance of circMETTL3 in the retina remain to be further investigated.

Therefore, the current study is designed to explore the biological function of circMETTL3 in the AMD, as well as the possible underlying mechanism involved. Our study uncovered that circMETTL3, miR‐100, and BMPR2 play important roles in H_2_O_2_‐induced ARPE‐19 cells. These findings suggested that circMETTL3, miR‐100, and BMPR2 are promising therapeutic targets for the treatment of AMD.

## 2. Materials and Methods

### 2.1. Cell Culture and Treatment

The ARPE‐19 cells (American Type Culture Collection, Manassas, VA, USA) were maintained in DMEM/F12 plus 10% FBS and 1% penicillin/streptomycin at 37°C with 5% CO_2_. The cells were incubated with 50, 250, and 500  μM H_2_O_2_ for 24  h to explore the effects of circMETTL3 level on senescence, oxidative stress, and DNA damage.

### 2.2. Cell Transfection

Shanghai Genepharm provided pcDNA3.1/circMETTL3, pcDNA3.1/BMPR2, and miR‐100 mimics/inhibitors. miR‐100‐5p inhibitor (5′‐CCACAAGUUCGGAUCUACGGGU‐3′)

NC inhibitor (5′‐AAGUACUAAUGUGUAGUACAAA‐3′). These vectors were transfected for 48 h using Lipofectamine 2000 (Invitrogen), and subsequent experiments were performed.

### 2.3. RT‐qPCR

Total RNA from ARPE‐19 cells was extracted using TRIzol reagent (Thermo Fisher, Waltham, MA, USA) and reverse transcribed into cDNA using M‐MLV Reverse Transcriptase (Thermo Fisher Scientific, Waltham, MA, USA). The qPCR was performed on SYBR Green Master Mix (Vazyme, Nanjing, Jiangsu, China). Gene expression quantification was performed using the 2^−ΔΔCt^ method. GAPDH served as an endogenous control.

### 2.4. CircRNA RNase R Resistance Analysis

The cells were treated with 3 μ/mg RNase R (Epicentre, WI, USA) and cultured at 37°C. Then, cells were purified using the RNeasy MinElute Cleaning Kit (Qiagen, Inc.). The experimental results were analyzed by RT‐qPCR.

### 2.5. RNA Sequencing Analysis

Total cell RNA was isolated using the RNeasy Mini Kit (Cat#74106, Qiagen). CircRNA sequencing analysis was conducted following experimental procedures at BioSCI Res (Shanghai, China).

### 2.6. Western Blotting

Western blot was performed following standard procedures described previously [17]. The membranes were then incubated overnight at 4°C with the primary antibodies (BMPR2, TJP1, BEST1, CTNNB1, KRT18, and GAPDH). After being washed with PBS, the membranes were incubated in appropriate secondary antibodies for 2 h. Finally, the membranes were visualized using an ECL chemiluminescence system.

### 2.7. MTT Assays

Cell proliferation was assessed with an MTT kit (Promega). Thereafter, cells were seeded in each well of a 24‐well plate and cultured in a medium containing 10% FBS for 2 weeks, with reagent change every 4 days during culture. Cell viability was determined by absorbance at 490 nm.

### 2.8. Luciferase Reporter Assays

The sequences of WT‐circMETTL3 or WT‐BMPR2 and its corresponding Mut‐circMETTL3 or Mut‐BMPR2 were synthesized by Sangon Bio‐technologies. Then, those vectors were co‐transfected with miR‐NC mimics or miR‐100 mimics into cells using the Lipofectamine 2000 reagent (Invitrogen). 48 h after transfection, luciferase activity was monitored using a dual luciferase reporting system (Promega).

### 2.9. RIP Assay

Cells were collected and cleaved with RIP lysis buffer. Whole‐cell extracts were then incubated with a RIP buffer containing magnetic beads bound to anti‐Ago2 or control IgG. Wash the beads with a washing buffer, then add protease K to digest the protein. Subsequently, immunoprecipitated RNA was isolated and analyzed by RT‐qPCR.

### 2.10. Senescence‐Associated (SA)‐β‐Gal Staining

The SA‐β‐Gal activity of ARPE‐19 cells was measured using the SA‐β‐gal staining kit (C0602, Beyotime, Shanghai). The treated cells were inoculated overnight in 6‐well plates, then fixed with 4% paraformaldehyde for 20 min and incubated overnight with the staining solution at 4°C. The image was captured using a light microscope.

### 2.11. Measurement of Intracellular ROS

The cells were incubated with 10 μmol/L DCFH‐DA at 37°C for 30 min. DCF fluorescence was detected by confocal microscopy at 488 nm excitation and 525 nm emission. The fluorescence intensity of DHE was analyzed by ImageJ.

### 2.12. Immunofluorescence (IF) Analysis

The cells were cultured on glass plates, then immobilized and infiltrated. After being closed in 5% BSA solution for 1 h and cleaned, it was incubated with γH2AX primary antibody overnight at 4°C. The samples were then washed in PBS and incubated with secondary antibody for 1 h, followed by incubation with DAPI (BD Biosciences, Franklin Lakes, NJ, USA) at room temperature for 5 min. IF was observed under a confocal laser scanning microscope (Leica SP5, Germany).

### 2.13. Statistical Analysis

All data were analyzed using SPSS Version 20.0 (IBM Corp.) and presented as the mean ± standard deviation (SD). Quantitative variables were analyzed by the Student’s *t*‐test between groups. One‐way ANOVA was used for multiple group comparisons. *p* < 0.05 was considered significant.

## 3. Results

### 3.1. CircMETTL3 Expression Was Downregulated in H_2_O_2_‐Triggered ARPE‐19 Cells

**FIGURE 1:**
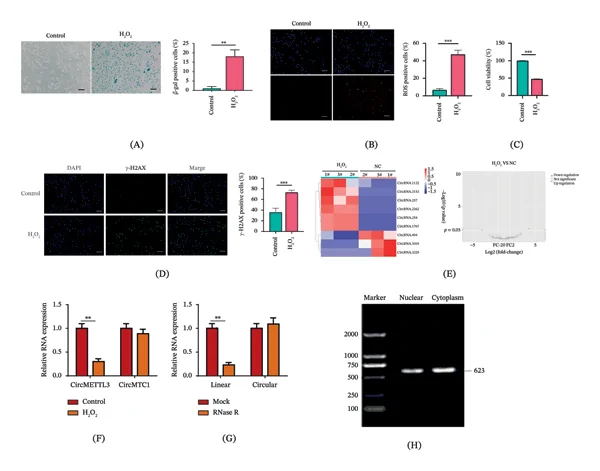
CircMETTL3 expression was downregulated in H_2_O_2_‐triggered ARPE‐19 cells. ARPE‐19 cells were treated with H_2_O_2_ and detected by SA‐β‐gal staining (A). The ROS level was detected in ARPE‐19 cells treated with H_2_O_2_ (B). MTT assay was utilized to assess cell viability in H_2_O_2_‐treated ARPE‐19 cells (C). IF assay was utilized to assess γH2AX expression in H_2_O_2_‐treated ARPE‐19 cells (D). A whole‐transcriptome sequencing was used to detect circRNA expression patterns in the H_2_O_2_ and NC models (*n* = 3). Heatmaps and volcano plots were used to visualize the differentially expressed circRNA (E). The expression of circMETTL3 and circMTCL1 were measured in H_2_O_2_‐treated ARPE‐19 cells (F). Expression levels of circMETTL3 and corresponding linear mRNA were determined using qPCR after RNase R treatment (G). The localization of circMETTL3 was analyzed using a nucleus cytoplasm separation assay (H). All experiments were performed with three biological replicates. Data are presented as mean ± SD. ^∗∗^ *p* < 0.01, ^∗∗∗^ *p* < 0.001.

To investigate the effect of H_2_O_2_ on circMETTL3 expression in ARPE‐19 cells, cells were treated with different concentrations of H_2_O_2_ for various time periods. The results showed that H_2_O_2_ significantly reduced circMETTL3 expression compared with the control group, with the lowest expression observed at a dose of 500 μM (Figure S1A). In addition, longer H_2_O_2_ treatment durations led to further decreases in circMETTL3 expression, with the lowest level occurring at 24 h (Figure S1B). Therefore, the 500 μM H_2_O_2_ treatment for 24 h was selected as the experimental model, which has also been used in previous studies [18, 19]. Figure 1A shows the enhanced SA‐β‐gal staining after H_2_O_2_ treatment in ARPE‐19 cells. Meanwhile, ARPE‐19 cells treated with H_2_O_2_ increased ROS production and decreased cell activity (Figure 1B, C). Moreover, γH2AX, an DNA damage indicator, was enhanced in H_2_O_2_‐triggered ARPE‐19 cells assessed by IF assay (Figure 1D). These results indicated the successful establishment of the cell model. Next, complete transcriptome sequencing was performed on H_2_O_2_‐treated and untreated ARPE‐19 cells to identify differentially expressed circRNAs. The results revealed that circMETTL3 and circMTCL1 levels were decreased in the H_2_O_2_‐stimulated group (Figure 1E). RT‐qPCR detected a decrease in circMETTL3 levels in H_2_O_2_‐triggered ARPE‐19 cells, while there was no significant change in circMTCL1 level, so we chose circMETTL3 for further study (Figure 1F). We further investigated circMETTL3 properties. First, the stability of circMETTL3 was evaluated by treating H_2_O_2_‐triggered ARPE‐19 cells with RNase R. The date indicated that circMETTL3 was resistant to RNase R treatment, while linear METTL3 was not (Figure 1G). In addition, circMETTL3 was distributed in both the cytoplasm and nucleus but was relatively abundant in the cytoplasm (Figure 1H).

### 3.2. CircMETTL3 Protected the Function of RPE In Vitro

**FIGURE 2:**
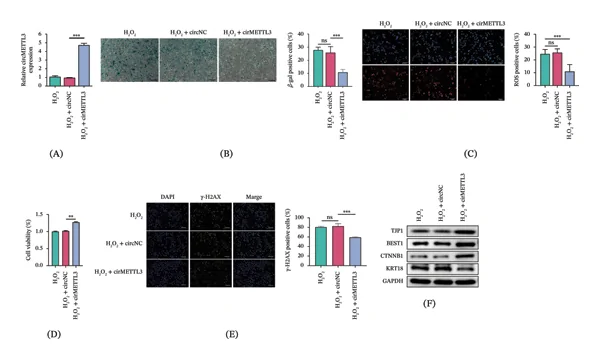
CircMETTL3 protected the function of RPE in vitro. ARPE‐19 cells were divided into H_2_O_2_, H_2_O_2_ + pcDNA3.1, and H_2_O_2_ + pcDNA3.1/circMETTL3 groups. The level of circMETTL3 was detected by RT‐qPCR (A). SA‐β‐gal staining experiments were conducted in the above groups (B). Measurement of intracellular ROS in the above groups (C). MTT assay was utilized to evaluate cell viability (D). The level of γH2AX was detected in H_2_O_2_, H_2_O_2_ + pcDNA3.1, and H_2_O_2_ + pcDNA3.1/circMETTL3 groups by IF assay (E). The expression of TJP1, BEST1, and CTNNB1 was measured by western blot (F). All experiments were performed with three biological replicates. Data are presented as mean ± SD. ns indicates no statistically significant difference; ^∗^ *p* < 0.05, ^∗∗^ *p* < 0.01, ^∗∗∗^ *p* < 0.001.

To evaluate the role of circMETTL3 in H_2_O_2_‐treated ARPE‐19 cells, ARPE‐19 cells were assigned to H_2_O_2_, H_2_O_2_ + pcDNA3.1, and H_2_O_2_ + pcDNA3.1/circMETTL3 groups. CircMETTL3 overexpression efficiency was verified by RT‐qPCR (Figure 2A). The addition of circMETTL3 decreased SA‐β‐gal staining and ROS production and accelerated cell viability in H_2_O_2_‐treated ARPE‐19 cells (Figure 2B–D). Moreover, the γH2AX level was decreased after circMETTL3 overexpression (Figure 2E). Furthermore, circMETTL3 increased the protein levels of RPE‐specific markers (TJP1, BEST1, and CTNNB1) while reducing the expression of the inflammatory marker KRT18 [20] (Figure 2F). These results suggested that the addition of circMETTL3 suppressed senescence, oxidative stress, and DNA damage in H_2_O_2_‐treated ARPE‐19 cells.

### 3.3. CircMETTL3 Serves as a ceRNA of miR‐100

**FIGURE 3:**
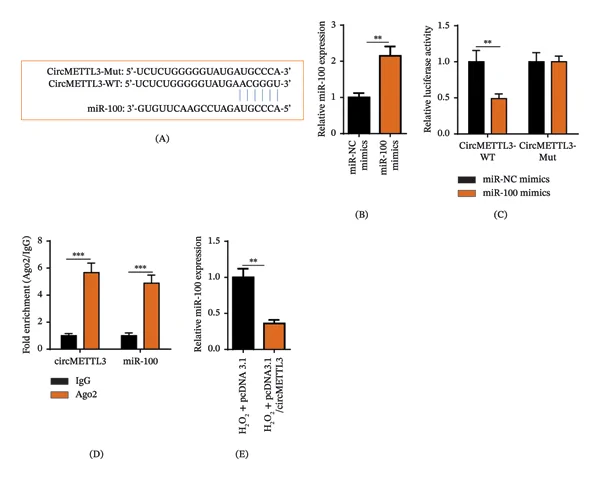
CircMETTL3 serves as a ceRNA of miR‐100. Bioinformatic analysis showed the binding sites between circMETTL3 and miR‐100 (A). miR‐100 expression was measured in ARPE‐19 cells transfected with miR‐NC mimics and miR‐100 mimics (B). Luciferase activity of cells was examined after co‐transfection with plasmids and mimics or the negative control (miR‐NC) (C). RIP assay was performed to assess the relationship between circMETTL3 and miR‐100 (D). miR‐100 expression was measured in ARPE‐19 cells transfected with H_2_O_2_ + pcDNA3.1 and H_2_O_2_ + pcDNA3.1/circMETTL3 (E). All experiments were performed with three biological replicates. Data are presented as mean ± SD. ^∗∗^ *p* < 0.01, ^∗∗∗^ *p* < 0.001.

The downstream target of circMETTL3 was investigated by CircInteractome (https://circinteractome.nia.nih.gov/). We identified a total of 9 downstream targets of circMETTL3 (see Supporting Table 1). Among them, miR‐100 has been reported in the literature to be involved in the progression of AMD. Specifically, H_2_O_2_ dose‐dependently reduced the viability of ARPE‐19 cells while concurrently upregulating both membrane‐bound and extracellular miR‐100 levels [21]. Treatment with miR‐100 antagonists exerted a cytoprotective effect against H_2_O_2_‐induced oxidative cell death. Therefore, we selected miR‐100 for further investigation [21]. The binding sites of miR‐100 and circMETTL3 are presented in Figure 3A. First, the upregulation efficiency of miR‐100 was analyzed by RT‐qPCR. Transfection results showed that miR‐100 mimics could increase the content of miR‐100 (Figure 3B). Then, it was found that the addition of miR‐100 restrained the luciferase activity of circMETTL3‐WT, while the luciferase activity of circMETTL3‐Mut remained unchanged (Figure 3C). RIP detection results revealed that circMETTL3 and miR‐100 were obviously enriched in the anti‐Ago2 group (Figure 3D). Moreover, circMETTL3 addition inhibited the miR‐100 level (Figure 3E). Overall, these findings confirmed that circMETTL3 was a sponge for miR‐100.

### 3.4. miR‐100 Reversed the Protective Effects of CircMETTL3 Against Senescence, Oxidative Stress, and DNA Damage in H_2_O_2_‐Treated ARPE‐19 Cells

**FIGURE 4:**
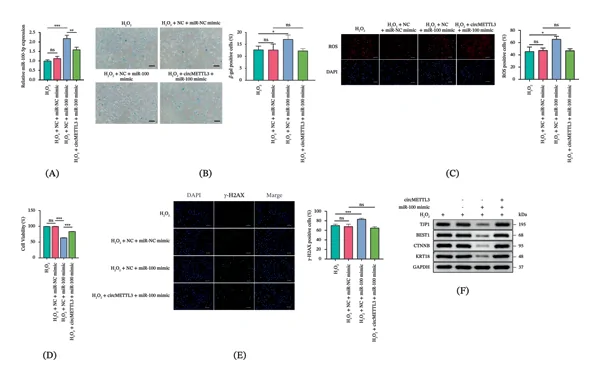
miR‐100 reversed the protective effects of circMETTL3 against senescence, oxidative stress, and DNA damage in H_2_O_2_‐treated ARPE‐19 cells. miR‐100 expression was measured in ARPE‐19 cells treated with H_2_O_2_ and transfected with miR‐NC mimic, miR‐100 mimic, or pcDNA3.1/circMETTL3. (A) ARPE‐19 cells were divided into H_2_O_2_, H_2_O_2_ + miR‐NC mimic + NC, H_2_O_2_ + miR‐100 mimic + NC, and H_2_O_2_ + miR‐100 mimic + circMETTL3 groups to measure SA‐β‐gal staining and ROS (B and C). MTT assay was utilized to measure the viability of ARPE‐19 cells (D). The level of γH2AX was detected by IF assay (E). The expression of TJP1, BEST1, CTNNB1, and KRT18 was measured by western blot (F). All experiments were performed with three biological replicates. Data are presented as mean ± SD. ns indicates no statistically significant difference; ^∗^ *p* < 0.05, ^∗∗^ *p* < 0.01, ^∗∗∗^ *p* < 0.001.

We subsequently investigated whether circMETTL3 exerts its protective effects in H_2_O_2_‐treated ARPE‐19 cells by regulating miR‐100. First, the expression of miR‐100 following miR‐100 mimic treatment was validated by RT‐qPCR. The miR‐100 mimic increased miR‐100 expression, whereas circMETTL3 overexpression reduced miR‐100 expression (Figure 4A). Overexpression of miR‐100 enhanced SA‐β‐gal staining and ROS production, while decreasing the viability of H_2_O_2_‐treated ARPE‐19 cells, thereby reversing the protective effects of circMETTL3 overexpression (Figure 4B–D). Moreover, miR‐100 mimic induced elevated γH2AX levels and decreased KRT18 expression, while increasing the expression of TJP1, BEST1, and CTNNB1, which reversed the effects of circMETTL3 overexpression (Figure 4E, F). In summary, miR‐100 reversed the alleviating effects of circMETTL3 overexpression on senescence, oxidative stress, and DNA damage in H_2_O_2_‐treated ARPE‐19 cells.

### 3.5. BMPR2 Presented as a Molecular Target of miR‐100

**FIGURE 5:**
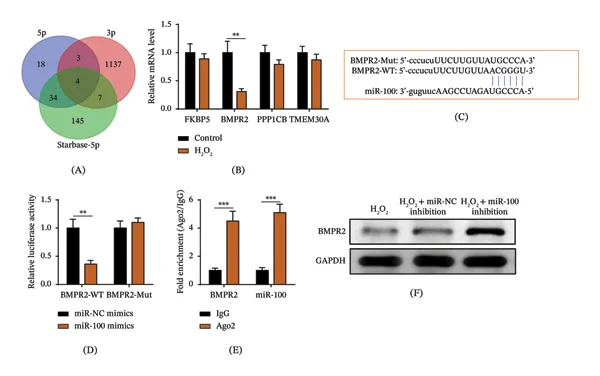
BMPR2 presented as a molecular target of miR‐100. Four downstream genes (FKBP5, BMPR2, PPP1CB, and TMEM30A) were obtained by Venn hybridization (A). The mRNA level of FKBP5, BMPR2, PPP1CB, and TMEM30A was measured by RT‐qPCR (B). The predictive binding sites between miR‐100 and BMPR2 were presented (C). The luciferase activities of WT‐BMPR2 and MUT‐BMPR2 in cells were evaluated after transfection with miR‐100 mimics (D). RIP assay was performed to assess the relationship between BMPR2 and miR‐100 (E). BMPR2 expression was measured in ARPE‐19 cells by western blot (F). All experiments were performed with three biological replicates. Data are presented as mean ± SD. ^∗∗^ *p* < 0.01, ^∗∗∗^ *p* < 0.001.

TargetScan was utilized to predict miR‐100‐3p or miR‐100‐5p downstream genes, which yielded 1151 and 60 genes, respectively. StarBase was performed to predict miR‐100‐5p genes, which yielded 197 genes. Four downstream genes (FKBP5, BMPR2, PPP1CB, and TMEM30A) were obtained by Venn crossing (Figure 5A), among which BMPR2 expression decreased in H_2_O_2_‐treated cells, while the expression of other genes showed no significant changes (Figure 5B). Based on this, BMPR2 was selected for further study. As established in Figure 5C, the 3′UTR of BMPR2 contains a site that binds to miR‐100. Moreover, the luciferase activity was decreased after co‐transfection with miR‐100 and BMPR2‐WT, while the luciferase activity was not affected after co‐transfection with BMPR2‐mut, indicating that miR‐100 could indeed bind to BMPR2 (Figure 5D). The RIP experiment further confirmed the results of 5D (Figure 5E). Furthermore, BMPR2 expression was enhanced in H_2_O_2_‐treated ARPE‐19 cells after miR‐100 deficiency (Figure 5F).

### 3.6. BMPR2 Overexpression Reversed the Damaging Effects of miR‐100 on H_2_O_2_‐Induced ARPE‐19 Cells

**FIGURE 6:**
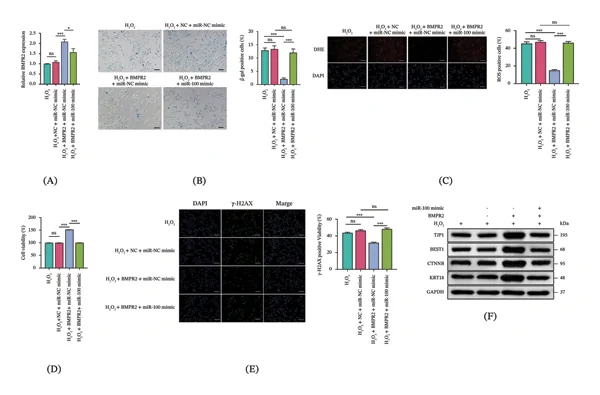
BMPR2 overexpression reversed the damaging effects of miR‐100 on H_2_O_2_‐induced ARPE‐19 cells. BMPR2 expression was detected by RT‐qPCRin ARPE‐19 cells treated with H_2_O_2_, and pcDNA3.1/BMPR2 or miR‐100 mimic (A). ARPE‐19 cells were divided into H_2_O_2_, H_2_O_2_ + miR‐NC mimic + NC, H_2_O_2_ + BMPR2 + miR‐NC mimic, and H_2_O_2_ + BMPR2 + miR‐100 mimic groups to measure SA‐β‐gal staining and ROS (B and C). MTT assay was performed to measure the viability of ARPE‐19 cells (D). The level of γH2AX was detected by IF assay (E). The expression of TJP1, BEST1, CTNNB1, and KRT18 was measured by western blot (F). All experiments were performed with three biological replicates. Data are presented as mean ± SD. ns indicates no statistically significant difference; ^∗^ *p* < 0.05, ^∗∗∗^ *p* < 0.001.

The expression of BMPR2 in H_2_O_2_‐treated ARPE‐19 cells after transfection with the BMPR2 plasmid vector was detected by PCR. As shown in Figure 6A, BMPR2 overexpression significantly increased BMPR2 expression, whereas miR‐100 mimic reduced BMPR2 expression. BMPR2 overexpression decreased the enhanced SA‐β‐gal staining and ROS production induced by H_2_O_2_, and increased cell viability, thereby reversing the damaging effects of miR‐100 (Figure 6B–D). In addition, BMPR2 overexpression reduced the levels of γH2AX and KRT18 in ARPE‐19 cells, while increasing the expression of TJP1, BEST1, and CTNNB1, which reversed the effects of miR‐100 (Figure 6E, F). In summary, BMPR2 overexpression reversed the aggravating effects of miR‐100 on senescence, oxidative stress, and DNA damage in H_2_O_2_‐treated ARPE‐19 cells.

## 4. Discussion

In the current study, we preliminarily identified differentially expressed circRNA in the H_2_O_2_ group and the NC group by high‐throughput sequencing. The low expression of circMETTL3 was further verified by the aging model of ARPE‐19 cells cultured with H_2_O_2_. Through functional experiments, we found that circMETTL3 could protect against oxidative stress, aging, and DNA damage stimulated by H_2_O_2_. The correlation between circMETTL3 and miR‐100 and miR‐100 and BMPR2 was confirmed by bioinformatics. Furthermore, miR‐100 overexpression exacerbated H_2_O_2_‐induced damage in ARPE‐19 cells, which reversed the protective effect of circMETTL3 on ARPE‐19 cells. Meanwhile, BMPR2 overexpression alleviated H_2_O_2_‐induced damage in ARPE‐19 cells, which reversed the damaging effect of miR‐100 on ARPE‐19 cells. Our results demonstrate the potential of the circMETTL3/miR‐100/BMPR2 as therapeutic targets for AMD.

With the development of bioinformatics and high‐throughput sequencing technology, the influence of circRNAs on biological and pathological processes has been paid more and more attention. By screening large samples, several circRNAs have been identified as reliable prognostic biomarkers and therapeutic targets, particularly in cancer. CircRNA has been reported to work in several ways. Since most circRNAs contain miRNA reaction elements, many studies have focused on the sponging effect of circRNAs on miRNAs. For example, circZNF532 [22] and circCOL1A2 [23] regulated retinal vascular dysfunction through different miRNAs in diabetic retinopathy. CircSV2b addition impeded the damage of oxidative stress by regulating miR‐5107 [24]. CircCCNB1 restrained cellular senescence via targeting miR‐449a to modulate CCNE2 [25]. CircITCH improved doxorubicin‐irritated cardiotoxicity via reducing DNA damage through modulation of miR‐330‐5p [26]. However, the role of circRNAs in equally important pathologic processes in AMD—cellular senescence, oxidative stress, and DNA damage—has not been well studied. It was observed that the circMETTL3 level was decreased in H_2_O_2_‐triggered ARPE‐19 cells. The addition of circMETTL3 reduced SA‐β‐gal staining and ROS and enhanced cell viability. Moreover, we found that circMETTL3 suppressed γH2AX expression and enhanced TJP1, BEST1, and CTNNB1 protein levels in H_2_O_2_‐triggered ARPE‐19 cells. Our data revealed that circMETTL3 improved H_2_O_2_‐induced senescence, oxidative stress, and DNA damage in ARPE‐19 cells.

Previous studies have demonstrated the involvement of ncRNAs in AMD modulation. For instance, miR‐21 was closely related to HIF‐1*α*/VEGF signaling pathway and promoted choroidal neovascularization formation in highly myopic guinea pigs [27]. MiR‐203a expression was decreased in AMD patients. MiR‐203a regulates the differentiation of RPE cells and thus affects the progression of AMD [28]. miR‐100, a target of circMETTL3, has been confirmed enhanced in H_2_O_2_‐triggered ARPE‐19 cells [21]. Consistent with previous findings, we also found that miR‐100 was elevated in H_2_O_2_‐stimulated ARPE‐19 cells. In addition, we also found that knockout of miR‐100 reduced SA‐β‐gal staining and ROS and promoted proliferation. miR‐100 inhibitor decreased the expression of γH2AX and KRT18, while increasing the levels of TJP1, BEST1, and CTNNB1 proteins. These results provide new insights into the role of miR‐100 in AMD. In order to further elucidate the molecular mechanism of miR‐100 in AMD, we identified BMPR2, the downstream target of miR‐100, using StarBase and TargetScan. BMPR2 has been increasingly recognized as a gene of relevance in AMD pathogenesis. A recent machine learning‐based diagnostic model identified BMPR2 as one of 10 key genes in the RPE/choroid complex that accurately distinguished AMD patients from normal controls, highlighting its potential as a diagnostic biomarker for AMD [29]. Moreover, Dhirachaikulpanich et al. reported that BMPR2 expression was decreased in aging human choroidal endothelial cells, suggesting that age‐related decline in BMPR2 may contribute to the vulnerability of the choroidal vasculature [30]. In the context of AMD, as our preprint has previously been published, it showed that the BMP signaling pathway, mediated by BMPR2, has been implicated in the regulation of RPE cell senescence and oxidative stress responses [31]. Consistent with the above observations, our study demonstrated that miR‐100 directly targets BMPR2 and that BMPR2 overexpression alleviates H_2_O_2_‐induced senescence, oxidative stress, and DNA damage in ARPE‐19 cells. These findings further support the notion that the miR‐100/BMPR2 axis represents a previously unrecognized regulatory mechanism contributing to RPE dysfunction in AMD. However, the precise downstream signaling pathways through which BMPR2 exerts its protective effects in RPE cells and whether BMPR2 restoration could ameliorate AMD‐like pathology in vivo warrant further investigation.

Despite the consistent and reproducible findings from our in vitro experiments, several limitations of this study should be acknowledged. First, all functional experiments were performed exclusively in H_2_O_2_‐treated ARPE‐19 cells, which, although widely accepted as a reliable in vitro model for oxidative stress‐induced retinal pigment epithelium injury, cannot fully recapitulate the complex pathological microenvironment of AMD in vivo. Second, due to the preliminary nature of this study and constraints in sample availability, we have not yet validated circMETTL3 expression in AMD animal models or clinical retinal specimens from AMD patients. Therefore, the translational relevance of our findings to human AMD remains to be established. Notably, the H_2_O_2_‐induced ARPE‐19 cell oxidative stress model is one of the most widely accepted and well‐characterized in vitro models for studying the pathogenesis of dry AMD, as extensively reported in the literature. We sincerely hope the reviewer can appreciate that this work is still at the preclinical mechanistic stage, and we have clearly outlined the necessary steps for future validation.

In conclusion, we identified the circMETTL3/miR‐100/BMPR2 network as a novel network involved in the etiology of AMD. Our results not only expand our understanding of the role of circRNAs in AMD but also identify a promising target for future AMD treatments.

**Nomenclature**
AMD: Age‐related macular degeneration
CircRNA: Circular RNA
ncRNAs: Noncoding RNAs
RT‐qPCR: Real‐time quantitative reverse transcription polymerase chain reaction
ROS: Reactive oxygen species
IF: Immunofluorescence
RPE: Retinal pigment epithelium
WB: Western blotting

## Author Contributions

Xiangyang Xin and Xin Zhao contributed to the study’s conception and design. Material preparation and data collection were performed by Liru Qin, Xinyue Liu, and Changhe Liu. Xin Zhao, Feng Ling, and Liru Qin performed the statistical analysis. Xin Zhao and Feng Ling carried out the analysis and interpretation. The first draft of the manuscript was written by Xiangyang Xin, Xin Zhao, and Feng Ling, and all authors commented on previous versions. Xiangyang Xin finished the critical revision and approved the final manuscript.

## Funding

This work was supported by Natural Science Foundation of Inner Mongolia Autonomous Region of China (2024LHMS08048) and Inner Mongolia Autonomous Region Science and Technology Innovation Guidance and Incentive Fund Project (CXYD2021BT05).

## Ethics Statement

The authors have nothing to report.

## Consent

The authors have nothing to report.

## Conflicts of Interest

The authors declare no conflicts of interest.

## Supporting Information

Additional supporting information can be found online in the Supporting Information section.

## Supporting information


**Supporting Information 1** Supporting Figure 1 Effect of H_2_O_2_ on circMETTL3 expression in ARPE‐19 cells. CircMETTL3 expression was detected by RT‐qPCR in ARPE‐19 cells treated with 0–500 μM H_2_O_2_ (A). CircMETTL3 expression was detected by RT‐qPCR in ARPE‐19 cells treated with 500 μM H_2_O_2_ for 0–24 h (B).

**Supporting Information 2** Supporting Table 1. Prediction and screening of microRNAs that may potentially bind to circMETTL3.

## Data Availability

The datasets used and/or analyzed during the current study are available from the corresponding author upon reasonable request.
