# Supplementary material for: CircMETTL3 Inhibits H2O2‐Induced Senescence, Oxidative Stress, and DNA Damage in ARPE‐19 Cells via miR‐100/BMPR2 Axis
Source: J Ophthalmol. 2026 Aug 20;2026:3194421. doi: 10.1155/joph/3194421 (PMC13492049; doi:10.1155/joph/3194421)
Supplement: Supplementary file 1 [file JOPH-2026-3194421-s002.docx]

**Supplementary Table 1. Prediction and screening of microRNAs that may potentially bind to circMETTL3.**

| **CircRNA  Mirbase ID** | **CircRNA (Top) - miRNA (Bottom) pairing** | **context+ score** | **context+ score percentile** |
| --- | --- | --- | --- |
| [hsa_circ_0031203](http://www.circbase.org/cgi-bin/singlerecord.cgi?id=hsa_circ_0031203) (5' ... 3')  [hsa-miR-1234](http://www.mirbase.org/cgi-bin/mirna_entry.pl?acc=hsa-miR-1234) (3' ... 5') | AACGCAUCAUUCGGACAGGCCGU                   \|\|\|\|\|\|\|      CACCCCACCCACCAGUCCGGCU | -0.3 | 96 |
| [hsa_circ_0031203](http://www.circbase.org/cgi-bin/singlerecord.cgi?id=hsa_circ_0031203) (5' ... 3')  [hsa-miR-1257](http://www.mirbase.org/cgi-bin/mirna_entry.pl?acc=hsa-miR-1257) (3' ... 5') | GACCCACCCUGGGAUAUUCACAU                  \|\|\|\|\|\|      CCAGUCUUGGGUAGUAAGUGA | -0.047 | 82 |
| [hsa_circ_0031203](http://www.circbase.org/cgi-bin/singlerecord.cgi?id=hsa_circ_0031203) (5' ... 3')  [hsa-miR-1322](http://www.mirbase.org/cgi-bin/mirna_entry.pl?acc=hsa-miR-1322) (3' ... 5') | CAAAUCAACUGCAACGCAUCAUU                \|\|\|\|\|\|\|      GUCGUAGUCGUCGUAGUAG | -0.137 | 90 |
| [hsa_circ_0031203](http://www.circbase.org/cgi-bin/singlerecord.cgi?id=hsa_circ_0031203) (5' ... 3')  [hsa-miR-361-3p](http://www.mirbase.org/cgi-bin/mirna_entry.pl?acc=hsa-miR-361-3p) (3' ... 5') | AGAAUGUCUAAAUCU---CUGGGGGU           \|\|\|\|    \|\|\|\|\|\|\|     UUUAGUCUUAGUGUGGACCCCCU | -0.341 | 96 |
| [hsa_circ_0031203](http://www.circbase.org/cgi-bin/singlerecord.cgi?id=hsa_circ_0031203) (5' ... 3')  [hsa-miR-485-3p](http://www.mirbase.org/cgi-bin/mirna_entry.pl?acc=hsa-miR-485-3p) (3' ... 5') | UCUAAAUCUCUGGGGGUAUGAAC                \|\|\|\|\|\|   UCUCUCCUCUCGGCACAUACUG | -0.039 | 74 |
| [hsa_circ_0031203](http://www.circbase.org/cgi-bin/singlerecord.cgi?id=hsa_circ_0031203) (5' ... 3')  [hsa-miR-567](http://www.mirbase.org/cgi-bin/mirna_entry.pl?acc=hsa-miR-567) (3' ... 5') | GAGAUGCGCAGGCUCAACAUACC                \|\|\|\|\|\|\|  CAAGACAGGACCUUCUUGUAUGA | -0.018 | 82 |
| [hsa_circ_0031203](http://www.circbase.org/cgi-bin/singlerecord.cgi?id=hsa_circ_0031203) (5' ... 3')  [hsa-miR-100](http://www.mirbase.org/cgi-bin/mirna_entry.pl?acc=hsa-miR-100) (3' ... 5') | UCUCUGGGGGUAUGAACGGGUAG                 \|\|\|\|\|\|    GUGUUCAAGCCUAGAUGCCCAA | -0.279 | 86 |
| [hsa_circ_0031203](http://www.circbase.org/cgi-bin/singlerecord.cgi?id=hsa_circ_0031203) (5' ... 3')  [hsa-miR-99a](http://www.mirbase.org/cgi-bin/mirna_entry.pl?acc=hsa-miR-99a) (3' ... 5') | UCUCUGGGGGUAUGAACGGGUAG                 \|\|\|\|\|\|    GUGUUCUAGCCUAGAUGCCCAA | -0.279 | 85 |
| [hsa_circ_0031203](http://www.circbase.org/cgi-bin/singlerecord.cgi?id=hsa_circ_0031203) (5' ... 3')  [hsa-miR-99b](http://www.mirbase.org/cgi-bin/mirna_entry.pl?acc=hsa-miR-99b) (3' ... 5') | UCUCUGGGGGUAUGAACGGGUAG                 \|\|\|\|\|\|    GCGUUCCAGCCAAGAUGCCCAC | -0.282 | 86 |
